# Flow Diversion vs. Stent-Assisted Coiling in the Treatment of Intradural Large Vertebrobasilar Artery Aneurysms

**DOI:** 10.3389/fneur.2022.917002

**Published:** 2022-06-10

**Authors:** Qiaowei Wu, Chunxu Li, Shancai Xu, Chunlei Wang, Zhiyong Ji, Jingtao Qi, Yuchen Li, Bowen Sun, Huaizhang Shi, Pei Wu

**Affiliations:** Department of Neurosurgery, The First Affiliated Hospital of Harbin Medical University, Harbin, China

**Keywords:** vertebrobasilar artery, large aneurysms, flow diverter, stent-assisted coiling, complications

## Abstract

**Objective:**

To compare the safety, angiographic, and long-term clinical outcomes of intradural large vertebrobasilar artery (VBA) aneurysms following flow diversion (FD) or conventional stent-assisted coiling (SAC).

**Methods:**

We performed a retrospective study of 66 consecutive patients with intradural large VBA aneurysms between 2014 and 2021 who underwent FD or SAC. Patients' characteristics, postprocedural complications, and clinical and angiographic outcome details were reviewed.

**Results:**

A total of 66 intradural large VBA aneurysms were included, including 42 (63.6%), which were treated with SAC (SAC group) and 24 (36.4%), which were treated with FD (FD group). Clinical follow-up was obtained at the median of 24.0 [interquartile range (IQR) 12.0–45.0] months, with 34 (81.0%) patients achieved the modified Rankin Scale (mRS) ≤ 2 in the SAC group and 21 (87.5%) patients in the FD group. Thirteen (19.7%) patients experienced neurological complications, of which 9 (13.6%) patients first occurred during the periprocedural phase and 4 (6.1%) patients first occurred during follow-up. The overall complication rate and periprocedural complication rate were both higher in the SAC group, but did not reach statistical significance (23.8 vs. 12.5%, *P* = 0.430; 16.7 vs. 8.3%, *P* = 0.564). The mortality rates were similar between the groups (11.9 vs. 12.5%). Angiographic follow-up was available for 46 patients at the median of 7 (IQR 6–14) months, with a numerically higher complete occlusion rate in the SAC group (82.1 vs. 55.6%, *P* = 0.051) and similar adequate aneurysm occlusion rates between the groups (85.7 vs. 83.3%, *P* = 1.000). In the multivariate analysis, ischemic onset (*P* = 0.019), unilateral vertebral artery sacrifice (*P* = 0.008), and older age (≥60 years) (*P* = 0.031) were significantly associated with complications.

**Conclusion:**

There was a trend toward lower complication rate and lower complete occlusion rate for intradural large VBA aneurysms following FD as compared to SAC. FD and SAC have comparable mortality rates and favorable outcomes. Ischemic onset, unilateral vertebral artery sacrifice, and older age could increase the risk of complications.

## Introduction

Intradural large (≥10 mm) vertebrobasilar artery (VBA) aneurysms are a very challenging subtype for physicians among the overall cerebral aneurysms, with a greater tendency to rupture, poorer natural history, and poorer outcomes compared with small aneurysms and anterior circulation aneurysms (1–3). Currently, there is no standard of care for the treatment of VBA aneurysms. The anatomy location and pathological feature of VBA aneurysms limit open surgical options, including clipping, wrapping, vessel occlusion, and bypass, which are often associated with high morbidity and mortality rates (4, 5). Stent-assisted coiling (SAC) has been increasingly used for treating such aneurysms, which can avoid the extensive surgical invasion and cranial nerve deficits associated with open surgery (6–8). However, due to the fusiform/dissecting morphology and relationship with perforating vessels, SAC of VBA aneurysms was still associated with a high rate of complication in published studies (6, 8, 9). In recent years, flow diversion (FD) has provided an optional method in treating cerebral aneurysms, but there are also potential risks in treating posterior circulation aneurysms (10, 11). Intradural large VBA aneurysms are rare and the different outcomes regarding the occlusion rate and the benefits/risks of FD vs. SAC have not been well evaluated. Therefore, we compared the postprocedural complications and long-term outcomes associated with FD and SAC for the treatment of intradural large VBA aneurysms.

## Materials and Methods

### Study Design and Patients

This study was a single-center retrospective study to compare the safety, angiographic, and long-term clinical outcomes of intradural large VBA aneurysms following FD or conventional SAC. The major criterion for inclusion in this study was aneurysms arising from the arterial segment extending from the origin of the intradural vertebral artery (VA) up to the origin of the superior cerebellar artery and measuring ≥10 mm in diameter. Aneurysms involving the extradural segment of VA, aneurysms arising from the branch vessel of VBA, basilar tip, and posterior cerebral artery aneurysms were excluded. Patients complicated by other treated anterior circulation aneurysms were also excluded.

Between January 2014 and October 2021, a total of 66 patients with intradural large VBA aneurysms, who underwent FD or SAC treatment, were consecutively enrolled in this study. The institutional review board of the First Affiliated Hospital of Harbin Medical University approved this retrospective study and a written informed consent was obtained from all the patients before the procedure.

### Endovascular Procedure

For patients with unruptured aneurysms, clopidogrel (75 mg/day) and aspirin (100 mg/day) were orally administrated at least 3–5 days before the endovascular procedure. After at least 3 days of administration of oral dual antiplatelet agent, thromboelastography (TEG) was used to study the platelet function in all the patients. The desired range of platelet inhibition was defined as arachidonic acid inhibition >50% and ADP inhibition >30%. If the TEG result did not archive the desired range, the clopidogrel (75 mg/day) would be changed to ticagrelor (90 mg/twice daily). For patients with ruptured aneurysms, aneurysms were treated within 24 h after admission, and tirofiban or a loading dose of 300mg clopidogrel and 300mg aspirin were administrated.

The treatment modalities (FD or SAC) were decided by the neurointerventionists (with >10 years of experience in neurointerventional surgery), depending on patient preferences and various anatomic factors, including aneurysm morphology and incorporation of major perforating branches of VBA and VA dominance. All the procedures were performed under general anesthesia and systemic heparinization. Three-dimensional (3D) rotational angiography was performed after the guiding catheter placement. The 3D reconstruction was then performed to determine the optimal work projection, measure the diameter of aneurysm and parent artery, and determine the stent size. Patients who underwent the FD treatment in this study were treated with Pipeline embolization device (Covidien, Irvine, California, USA). A Marksman microcatheter (Covidien, Irvine, California, USA) was used to deploy the FD. Postprocedural multiangle unsubtracted angiogram and VasoCT were performed to evaluate the FD expansion. Balloon angioplasty or in-stent massage with microcatheter and micro guidewire was performed in case of improper FD expansion. For patients who underwent the SAC treatment, aneurysms were coiled with stent assisted by the Low-profile Visualized Intraluminal Support (LVIS) (MicroVention-Terumo, California, USA), Enterprise (Codman Neurovascular, Massachusetts, USA), or Solitaire (ev3, Irvine, California, USA). Coils were packed until satisfactory aneurysm occlusion was achieved and/or additional packing was not possible. Clopidogrel (75 mg/day) and aspirin (100 mg/day) were continued for at least 3 months (6 months for patients treated with FD) after the procedure and aspirin (100 mg/day) was maintained indefinitely.

### Collected Data

Medical charts and imaging data were reviewed to identified patient age, sex, hypotension, diabetes mellitus, smoking, alcohol abuse, the Hunt–Hess grades for ruptured aneurysms, aneurysm size, aneurysm morphology, aneurysm location, incorporation of a branch artery, stent types, coil usage, postprocedural morbidity and mortality, follow-up imaging results, follow-up complications, clinical outcomes, and need for retreatment. Postprocedural complications (including thromboembolic events, hemorrhagic events, or new cranial nerve deficits) were diagnosed clinically as new neurologic deficits or changes in the level of consciousness or on CT/MR performed on patients of sudden neurologic deficits.

Clinical outcomes were collected at discharge and at follow-up (3, 6 months, and annual follow-up postprocedurally), which were evaluated by the modified Rankin Scale (mRS) score. The mRS score ≤ 2 was defined as a favorable outcome. Angiographic follow-up was scheduled at 6 months and 1–2 years postprocedurally. The Raymond–Roy grading scale was used to evaluate the angiographic outcomes (12). Grade 1 of the Raymond–Roy grading scale was defined as complete aneurysm occlusion and Grade 1/2 of the Raymond–Roy grading scale was defined as an adequate aneurysm occlusion.

### Statistical Analysis

Normally distributed continuous variables were presented as mean ± SD, while nonnormally distributed continuous variables were presented as median and interquartile range (IQR). Data were presented as numbers followed by percentages for qualitative variables. Analysis of variables between the two groups was carried out by using the Mann–Whitney *U* test or independent sample *t*-test for continuous variables and the chi-squared test or the Fisher's exact test for qualitative variables. The univariate and multivariate Cox regression analysis was performed to further identify the risk factors for postprocedural complications. Variables significant on the univariate analysis (*P* <0.05) were subjected to the multivariate analysis. A *P*-value <0.05 (two-sided) was considered to indicate statistical significance. Statistical analysis was performed using the SPSS version 22.0 software (IBM SPSS Incorporation, Chicago, Illinois, USA).

## Results

### Patient Characteristics

A total of 66 patients harboring 66 intradural large VBA aneurysms, who underwent FD or SAC treatment, were included in this study. The cohort comprised 24 (37.0%) females and 42 (63.0%) males, with a mean age of 55.4 ± 9.0 (range: 32–79) years. For 12 patients with ruptured aneurysms, 4 (33.3%) patients were the Hunt–Hess grade 1, 5 (41.7%) patients were the Hunt–Hess grade 2, and 3 (25%) patients were the Hunt–Hess grade 3. Forty-three (65.2%) aneurysms were originated from the intradural vertebral artery, 15 (22.7%) aneurysms were originated from the basilar artery, and 8 (12.1%) aneurysms were originated from the vertebrobasilar artery junction. The maximal median diameter of the aneurysms was 13.2 (IQR 11.0–12.2) (range: 10.0–29.8) mm in the cohort. FD was administrated in 24 (36.4%) patients (FD group) and 42 (63.6%) patients underwent SAC treatment (SAC group). The main baseline characteristics of the SAC and FD groups are as follows: mean age was 56.7 ± 9.0 years in the SAC group and 53.2 ± 8.9 years in the FD group, median aneurysm size was 13.2 (IQR 11–16) mm and 13.3 (IQR 11.8–21) mm, respectively, anterior inferior cerebellar artery (AICA) or posterior inferior cerebellar artery (PICA) was involved in 10 (23.8%) aneurysms in the SAC group and 3 (12.5%) aneurysms in the FD group, and all 12 (28.6%) ruptured aneurysms were treated with SAC. All the parent arteries were normal, without stenosis, tortuosity, or difficult vascular access. In addition to the rate of aneurysm rupture cases (*P* = 0.010), no other obvious differences of baseline characteristics of patients and aneurysms were observed between the groups and detailed characteristics are given in Table 1.

**Table 1:** Baseline characteristics of patients and aneurysms.

**Characteristics**	**FD group (*n =* 24)**	**SAC group (*n =* 42)**	***P-*value**
Male, *n* (%)	17 (70.8)	25 (59.5)	0.358
Age (years) (m ± SD)	53.2 ± 8.9	56.7 ± 9.0	0.126
Risk factors, *n* (%)			
Hypertension	14 (58.3)	23 (54.8)	0.779
Diabetes mellitus	1 (4.2)	3 (7.1)	1.000
Smoking	9 (37.5)	11 (26.2)	0.336
Alcohol abuse	7 (29.2)	15 (35.7)	0.587
Presented with ischemic symptoms, *n* (%)	7 (29.2)	8 (19.0)	0.345
Presented with hemorrhage, *n* (%)	0	12 (28.6)	0.010
Aneurysm location, *n* (%)			0.128
VBJ	5 (20.8)	3 (7.1)	
BA	3 (12.5)	12 (28.6)	
VA	16 (66.7)	27 (64.3)	
Aneurysm size (mm) (IQR)	13.3 (11.8, 21.0)	13.2 (11.0, 16.0)	0.292
Aneurysm size classification, *n* (%)			0.845
Large (10-15 mm)	16 (66.7)	27 (64.3)	
Very large or giant (>15 mm)	8 (33.3)	15 (35.7)	
Side branch involved, *n* (%)	3 (12.5)	10 (23.8)	0.430

*FD, flow diversion; SAC, stent-assisted coiling; SD, standard deviation; BA, basilar artery; VBJ, vertebrobasilar junction; VA, vertebral artery; IQR, interquartile range*.

### Procedures

Of the 42 aneurysms in the SAC group, 26 (61.9%) aneurysms were treated with overlapping stent-assisted coiling and 16 (38.1%) aneurysms were treated with single stent-assisted coiling. The median number of coils used in the SAC group was 8.5 (IQR 5.0–12.3). Twenty-six FDs were used to treat 24 aneurysms, of which 1 (4.2%) aneurysm was treated with 3 overlapping FDs. All the aneurysms in the FD group were treated with FD alone without adjunctive coiling. Unilateral vertebral artery sacrifice was performed in 5 (7.6%) patients, of which 3 (7.1%) patients in the SAC group and 2 (8.3%) patients in the FD group. The details of the procedure-related data are shown in Table 2.

**Table 2:** Endovascular procedure details.

**Procedure details**	**Number of patients**
FD	*n =* 24 patients
Single PED	22
Single PED and unilateral vertebral artery sacrifice	1
PED ×3 and unilateral vertebral artery sacrifice	1
SAC	*n =* 42 patients
Single EP assisted coiling	9
EP ×2 assisted coiling	3
EP + LVIS assisted coiling	17
EP + LVIS assisted coiling and unilateral vertebral artery sacrifice	1
EP + LVIS ×2 assisted coiling	1
EP + LVIS ×2 assisted coiling and unilateral vertebral artery sacrifice	1
EP ×2 + LVIS assisted coiling	1
Single LVIS assisted coiling	5
Single LVIS assisted coiling and unilateral vertebral artery sacrifice	1
LVIS ×2 assisted coiling	2
Solitaire assisted coiling	1

*FD, flow diversion; PED, pipeline embolization device; SAC, stent-assisted coiling; EP, enterprise stent; LVIS, low-profile visualized intraluminal support*.

### Comparison of Clinical Outcomes

All the patients' final clinical outcomes were available for analysis, with the median clinical follow-up time of 24.0 (IQR 12.0–45.0) months, including 35.5 (IQR 23.0–61.0) months in the SAC group and 13.0 (IQR 9.0–20.0) months in the FD group (*P* <0.001). As shown in Table 3, a total of 13 (19.7%) patients experienced postprocedural neurological complications, of which 9 (13.6%) patients first occurred during the periprocedural phase and 4 (6.1%) patients first occurred during follow-up. The overall complication rate and periprocedural complication rate were both higher in the SAC group, but did not reach statistical significance [23.8 vs. 12.5%, relative risk (RR) = 1.91, 95% CI: 0.58–6.25, *P* = 0.430; 16.7 vs. 8.3%, RR = 2.00, 95% CI: 0.45–8.87, *P* = 0.564]. A total of 8 (12.1%) patients died during the follow-up. The mortality rates were similar between the groups (11.9 vs. 12.5%, *P* = 1.000). The proportion of favorable outcomes (mRS ≤ 2) at last follow-up were also similar (81.0 vs. 87.5%, *P* = 0.731). We found no differences in the rates of overall postprocedural ischemic complications (19.0 vs. 12.5%, *P* = 0.731) or hemorrhagic complications (4.8 vs. 4.2%, *P* = 1.000) between SAC and FD treatments. The details of the treatment and angiographic outcome of patients are shown in Table 3.

**Table 3:** Treatment and angiographic outcome of patients.

**Characteristics**	**SAC group**	**FD group**	**RR (95% CI)**	***P*-value**
Number of patients with clinical FU	42	24	-	-
Median clinical FU (m) (IQR)	35.5 (23.0–61.0)	13.0 (9.0–20.0)	-	P <0.001
Overall complications, *n* (%)*	10 (23.8)	3 (12.5)	1.91 (0.58–6.25)	0.430
Death	5 (11.9)	3 (12.5)	0.95 (0.25–3.64)	1.000
Ischemic complications	8 (19.0)	3 (12.5)	1.52 (0.45–5.21)	0.731
Hemorrhage	2 (4.8)	1 (4.2)	1.14 (0.11–11.95)	1.000
Periprocedural complications, *n* (%)^†^	7 (16.7)	2 (8.3)	2.00 (0.45–8.87)	0.564
Death	1 (2.4)	0	-	1.000
Ischemic complications	5 (11.9)	2 (8.3)	1.43 (0.30–6.81)	0.970
Hemorrhage	2 (4.8)	1 (4.2)	1.14 (0.11–11.95)	1.000
Complications during FU, *n* (%)^†^	7 (16.7)	3 (12.5)	1.33 (0.38–4.68)	0.922
Death	4 (9.5)	3 (12.5)	0.76 (0.19–3.12)	1.000
Ischemic complications	7 (16.7)	3 (12.5)	1.33 (0.38–4.68)	0.922
Hemorrhage	0	1 (4.2)	-	0.364
mRS at discharge, *n* (%)				0.758
0–2	38 (90.5)	23 (95.8)	0.94 (0.83–1.07)	
3–6	4 (9.5)	1 (4.2)	2.29 (0.27–19.30)	
mRS at last FU, *n* (%)				0.731
0–2	34 (81.0)	21 (87.5)	0.93 (0.75–1.14)	
3–6	8 (19.0)	3 (12.5)	1.52 (0.45–5.21)	
Number of patients with angiographic FU	28	18	-	-
Median angiographic FU (m) (IQR)	10.5 (6.0–24.0)	6.5 (6.0–10.0)	-	0.061
Aneurysm angiographic finding, *n* (%)				
Complete occluded	23 (82.1)	10 (55.6)	1.48 (0.95–2.31)	0.051
Adequate occluded	24 (85.7)	15 (83.3)	1.03 (0.80–1.33)	1.000
In-stent stenosis/thrombosis	0	2 (11.1)	-	0.148
Retreatment	2 (7.1)	0	-	0.513

*SAC, stent-assisted coiling; FD, flow diverter; RR, relative risk; CI, confidence interval; FU, follow-up; IQR, interquartile range; mRS, modified Rankin Scale*. ^*^One patient in the FD group experienced the ischemic and hemorrhagic events. ^†^*Four patients in the SAC group and one patient in the FD group experienced two ischemic events, one within periprocedural period and one during the follow-up; One patient in the FD group experienced the ischemic and hemorrhagic events within periprocedural period, and ischemic and hemorrhagic events during the follow-up*.

### Complications

Among the 10 patients with postprocedural complications in the SAC group, one patient presented with one-sided motor weakness on the day after procedure and died due to the acute cerebral infarctions during the follow-up. Two patients presented with dysphagia postprocedurally and both died related to acute cerebral infarctions during the follow-up. Two patients experienced the persistent dizziness, of which 1 patient died due to the acute cerebral infarctions during the follow-up and the other patient improved after medication with the final mRS score of 1. One patient with ruptured aneurysms died due to the rerupture 3 days after the procedure. Two patients experienced one-sided motor weakness during the follow-up at 7 and 24 months, respectively, with the final mRS scores of 3 and 4, respectively. One patient experienced dysphagia and two-sided motor weakness on the day after procedure. The following brain MRI demonstrated the acute cerebral infarctions and the final mRS score was 4. Periprocedural symptomatic cerebellar hemorrhage occurred in one patient and the final mRS scores was 0.

**Figure 1:**
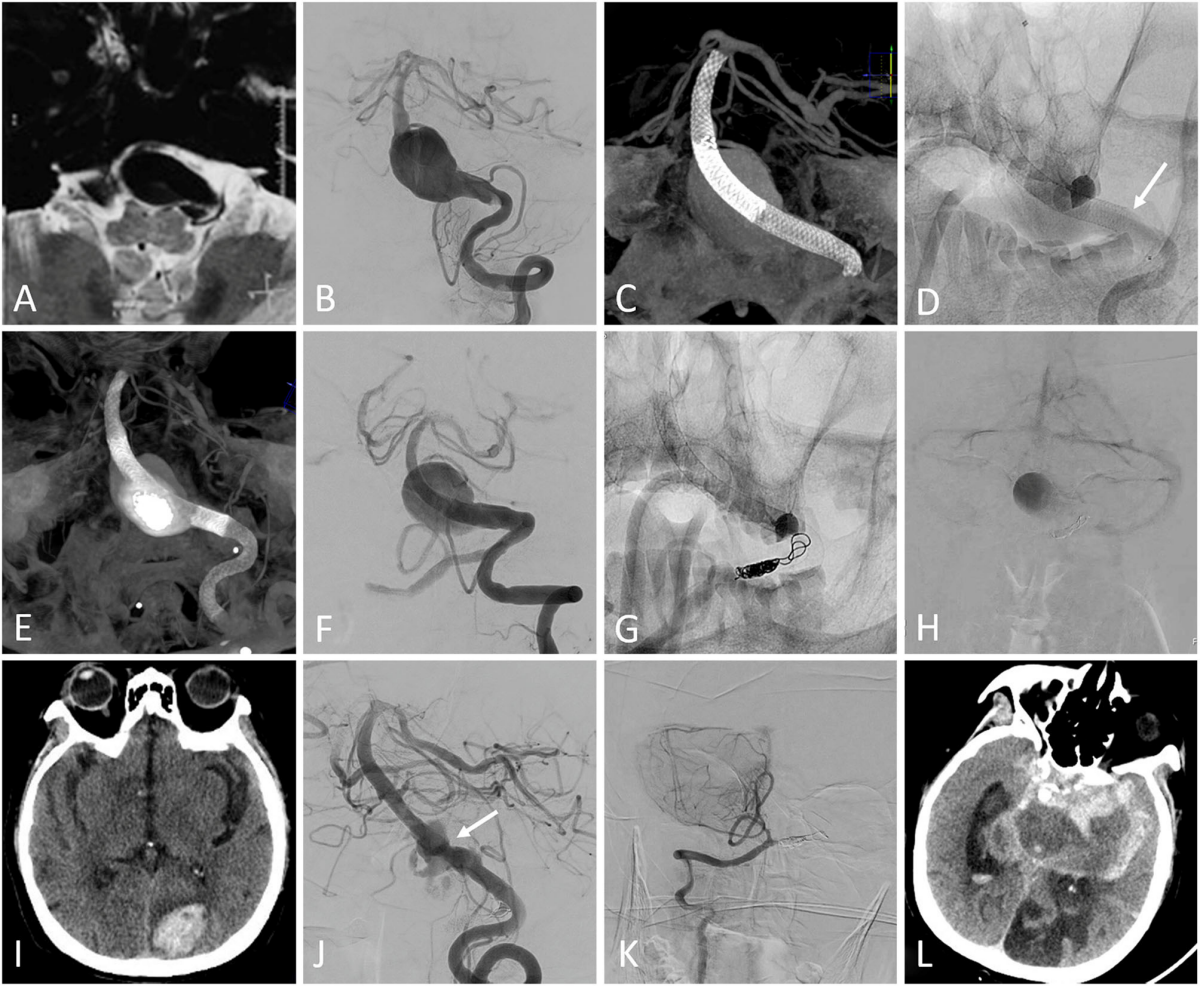
**(A)** MRI showed a large lesion extending from the medulla oblongata to the pons. **(B)** Pretreatment digital subtraction angiography (DSA) demonstrated a basilar artery aneurysm. **(C)** Two overlapping Pipeline embolization devices (PEDs) were deployed without adjunctive coiling. **(D)** The second PED shortened after the complete deployment (the arrow showed the proximal end of the second PED). **(E)** A third PED was deployed. **(F)** The DSA showed the patency of the parent artery. **(G)** The contralateral vertebral artery was occluded using coils and the blood flow detained to the venous phase was detected on postprocedural DSA **(H)**. The patient experienced right-sided motor weakness on the day after the procedure and the following CT did not show any hemorrhage. The motor weakness temporarily resolved after the administration of tirofiban and low-molecular weight heparin, but the left occipital lobe intraparenchymal hemorrhage was detected on CT 2 weeks after the procedure **(I)**. The patient experienced left-sided motor weakness 18 months after the procedure and the follow-up DSA showed the partial residual of the aneurysm, with mild in-stent stenosis (arrow) **(J)**. The right posterior inferior cerebellar artery remained patency **(K)**. The aneurysm ruptured 2 days after the angiography **(L)** and the patient finally died.

In the FD group, one patient experienced acute cerebral infarctions on the day after the procedure, followed by cerebral hemorrhage: the mRS score at discharge was 4. The patient experienced another cerebral infarction during the follow-up and finally died due to the aneurysm rupture (Figure 1). One patient developed acute parent artery occlusion directly after the procedure and the intra-arterial urokinase was then performed. The patient died due to acute cerebral infarctions during the follow-up, though the mRS score at discharge was 0. The other one patient died due to the acute in-stent thrombosis 5 months after the procedure (Table 3).

### Comparison of Angiographic Outcomes

Angiographic outcomes were available for 46 patients at the median follow-up time of 7.0 (IQR 6.0–14.0) months, including 10.5 (IQR 6.0–24.0) months in the SAC group and 6.5 (IQR 6.0–10.0) months in the FD group (*P* = 0.061). The overall complete occlusion rate was 71.7% (33/46). A numerically higher complete occlusion rate in the SAC group (82.1 vs. 55.6%, RR = 1.48, 95% CI: 0.95–2.31, *P* = 0.051), and similar adequate aneurysm occlusion rates between the groups (85.7 vs. 83.3%, *P* = 1.000) were found. Two (7.1%) aneurysms in the SAC group received the retreatment. One (5.6%) mild in-stent stenosis and one (5.6%) fatal in-stent thrombosis were found in the FD group.

### Factors Associated With Complications

**Figure 2:**
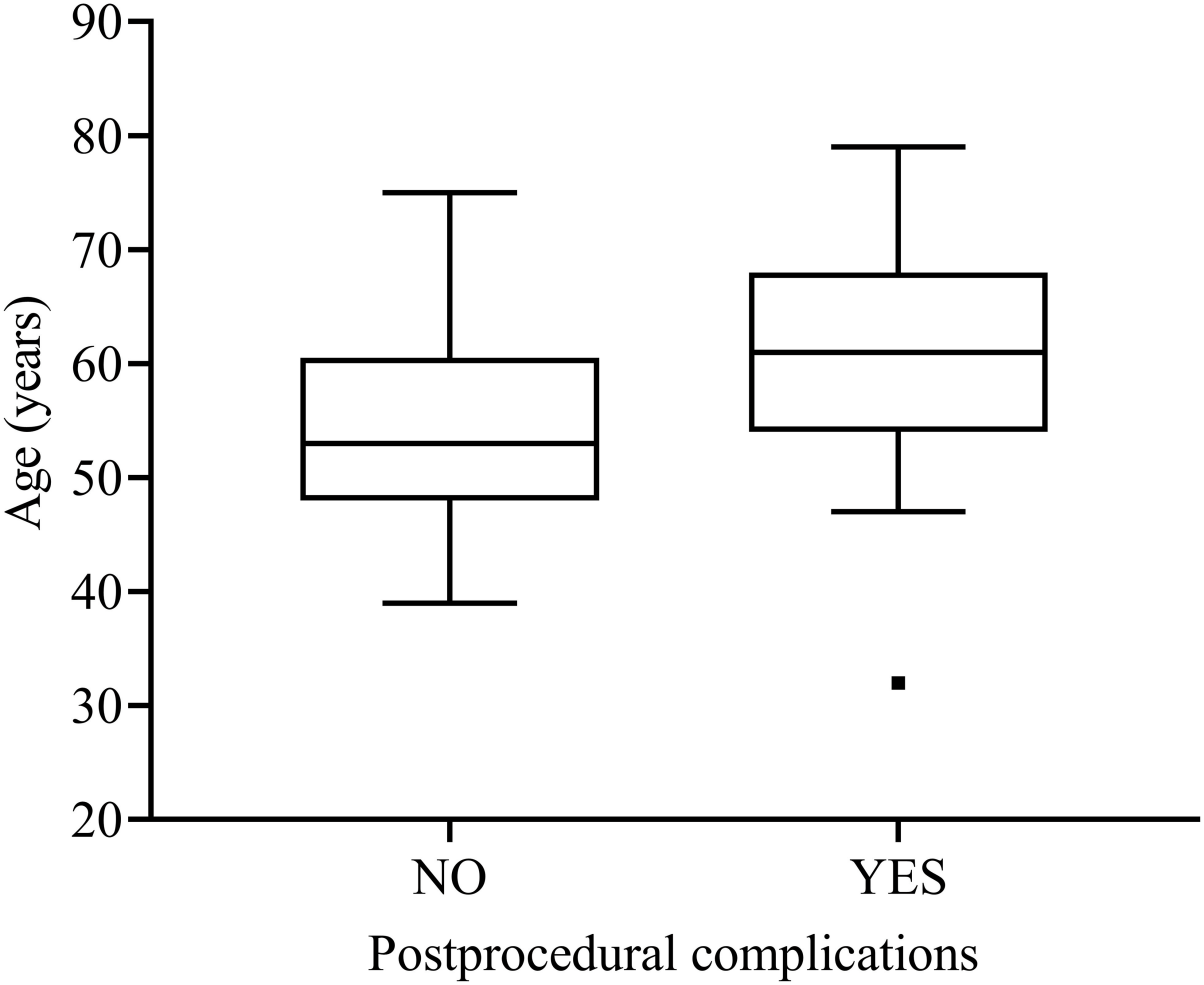
Box plot for age and postprocedural complications.

In the univariate Cox regression analysis, we found that older age (≥60 years old) (*P* = 0.012), aneurysms involved basilar artery (*P* = 0.002), patients with ischemic onset (*P* = 0.004), and unilateral vertebral artery sacrifice (*P* < .001) were related to postprocedural complications. In the multivariate Cox regression analysis, the following factors were associated with overall postprocedural complications statistically significantly: ischemic onset [hazard ratio (HR): 4.48, 95% CI: 1.29 to 15.61; *P* = 0.019], unilateral vertebral artery sacrifice (HR: 7.81, 95% CI: 1.72 to 35.53; *P* = 0.008), and older age (HR: 4.22, 95% CI: 1.14–15.67; *P* = 0.031) (Figure 2 and Table 4).

**Table 4:** The univariate and multivariate Cox regression analysis for postprocedural complications.

**Variable**	**Hazard ratio**	**95% confidence interval**	***P*-value**
**Univariate analysis**			
Age, (≥60 years)	4.53	1.40–10.21	0.012
Incorporation of the branch vessel	1.20	0.33–4.37	0.786
Sex (male)	0.92	0.30–2.82	0.885
Aneurysms involved basilar artery	7.65	2.10–28.00	0.002
Ruptured aneurysms	0.76	0.17–3.45	0.725
Hypertension	2.74	0.75–9.95	0.126
Ischemic onset	5.09	1.69–15.34	0.004
Unilateral vertebral artery sacrifice	11.65	3.07–44.20	<0.001
Diabetes mellitus	1.34	0.17–10.30	0.779
Smoking	0.40	0.09–1.79	0.229
Alcohol abuse	1.20	0.39–3.66	0.755
Flow diversion	0.56	0.15–2.03	0.373
Aneurysm size (>15 mm)	2.25	0.76–6.70	0.145
**Multivariate analysis**			
Age, (≥60 years)	4.22	1.14–15.67	0.031
Aneurysms involved basilar artery	2.12	0.46–9.86	0.337
Ischemic onset	4.48	1.29–15.61	0.019
Unilateral vertebral artery sacrifice	7.81	1.72–35.53	0.008

## Discussion

Intradural VBA aneurysms are relatively rare as compared to anterior circulation aneurysms. In the International Study of Unruptured Intracranial Aneurysms (ISUIA) (1), vertebrobasilar or posterior cerebral artery aneurysms account for only about 8% of all the aneurysms. However, such aneurysms presented a higher risk of rupture than aneurysms in other location and the diameter of aneurysm ≥10 mm was more likely to rupture than smaller ones. As reported by Mizutani et al. (13), once the rupture of VBA aneurysms occurred, if untreated, about 70% of patients subsequently underwent rerupture and about half of them subsequently died. Open surgical procedures, including trapping, clipping, and parent artery occlusion along with bypass, have been attempted to treat intradural VBA aneurysms in the past (14, 15). However, due to the limited surgical accessibility and relatively high mortality of open surgical procedures, endovascular reconstructive therapies, including SAC and FD, have been increasingly used. FD has been attempted to apply in the treatment of intradural large VBA aneurysms, but the results regarding the safety and efficacy remain controversial, as compared to SAC (7, 16, 17). We aimed to compare the treatment outcomes of FD and SAC for the treatment of intradural large VBA aneurysms.

Our results revealed a trend toward lower overall complication rate and periprocedural complication rate in the FD cohort. Intra-aneurysmal partial thrombosis is a common phenomenon in intradural large VBA aneurysms. The intra-aneurysmal mechanical manipulation may cause thrombus detachment from the aneurysm, which may cause the distal vessel occlusion or branch vessel occlusion, leading to ischemic complication or ischemia-reperfusion hemorrhage (7, 18, 19). In this study, all the patients in the FD group were treated with FD alone without adjunctive coiling. The nonintra-aneurysmal mechanical manipulation may associate with a lower complication rate in the FD group. A relatively high coil packing density in the SAC group may increase the risk of ischemic and hemorrhagic complications (20). In addition, the coil embolization in the SAC group may also increase the procedure time and endovascular mechanical manipulation, increasing the risk of vascular endothelial injury, atherosclerotic plaque detachment, and platelet aggregation (19).

This study demonstrated a complete occlusion rate of 55.6% for intradural large VBA aneurysms treated with FD, slightly lower than the complete occlusion rate for the overall posterior circulation aneurysm cohort, which was around 65% (10). Larger aneurysm size in our cohort could be a potential reason, which has been demonstrated to be associated with incomplete occlusion after FD treatment (21, 22). In addition, the results of this study showed a numerically higher complete occlusion rate in the SAC group (82.1 vs. 55.6%), but the adequate aneurysm occlusion rates were similar (85.7 vs. 83.3%). Published studies have reported the adjunctive coiling in patients with large or giant aneurysms treated with FD that could promote the thrombosis formation and aneurysm occlusion (23, 24). However, on one hand, the adjunctive coiling increased the cost of the procedure; on the other hand, the risks of procedure-related complication, procedure time, and fluoroscopy time also increased. Moreover, a relatively high coil packing density could also lead to the persistence of the mass effect of the aneurysms. In this study, no aneurysm in the FD group was treated with adjunctive coiling, which may be associated with a relatively low complete occlusion rate during follow-up. Furthermore, more than a half of patients in the SAC group underwent overlapping LVIS stents technique or LVIS-within-enterprise technique, which has been reported to be associated with less risk of aneurysm recurrence (25, 26). But, satisfactorily, the adequate aneurysm occlusion rates were similar between the groups, which were also similar to the results of meta-analysis by Domingo et al. on the treatment of intracranial aneurysms with FD and SAC (27).

We found that patients with ischemic symptoms before procedure were a risk factor of postprocedural complications in this study, which indicated that patient with ischemic onset still at risk of ischemic complication or ischemia-reperfusion hemorrhage after procedure. Similar result has been shown by Flemming et al. (28), who reported the natural history of vertebrobasilar nonsaccular aneurysms and found that the risk of recurrent cerebral infraction was 6.7% per year in patients presenting initially with ischemic infraction. They also found that the risk of cerebral infraction due to the target aneurysm increased from 2.7 at 1 year to 11.3% at 5 years and 15.9% at 10 years. In addition, this study revealed that unilateral vertebral artery sacrifice might increase the postprocedural complication rate. Due to both the vertebral arteries are converged at the vertebrobasilar junction and the occlusion site was located at the vertebrobasilar junction, we did not conduct the balloon occlusion test (BOT) on the premise of ensuring the patency of PICA. However, despite successful BOT, there was still a risk of delayed ischemic complications described by published studies and unpredicted hypoperfusion may be the possible reason (28, 29). After the occlusion, organized thrombus in the occluded artery may dislodge at any time due to the clot propagation. The gradual contraction of the occluded artery may eject the thrombus into the side branches and leads to delayed ischemic complication or ischemia-reperfusion hemorrhage (29). Older age (≥60 years) was associated with postprocedural complications in this study. In the ISUIA, older age has also been demonstrated to be the only risk factor of poor clinical outcome for patients who underwent surgical treatment (1). The intra-arterial mechanical manipulation during the procedure may cause the arterial injuries and the delayed endothelial healing may increase the risk of postprocedural ischemic events. Gennaro et al. (30) found that the impairment of reendothelialization was age dependent and aging could negatively regulate endothelial healing after injury. The comorbidities, such as atherosclerotic, and impairment of reendothelialization after endovascular procedure in the elderly may be associated with poor outcomes.

There are some limitations to this study. The sample size in this study was relatively small and the CIs are wide when analyzing the potential risk factors of overall complications, resulting in lower power for statistical analysis. In addition, 30.3% of patients were lost to angiographic follow-up, which might impact the evaluation of aneurysm healing. Also, due to the retrospective, nonrandomized design, the baseline data were not totally balanced and the potential bias (selection bias and so on) inherent to all the retrospective studies is unavoidable. Thus, a randomized controlled trial with large sample size would be of great interest.

## Conclusion

This study showed a trend toward lower complication rate and lower complete occlusion rate for intradural large VBA aneurysms following FD as compared to SAC. FD and SAC have comparable mortality rates, favorable outcomes, and adequate aneurysm occlusion rates. Moreover, ischemic onset, unilateral vertebral artery sacrifice, and older age could increase the risk of complications.

## Data Availability Statement

The raw data supporting the conclusions of this article will be made available by the authors, without undue reservation.

## Ethics Statement

The studies involving human participants were reviewed and approved by the Institutional Review Board of the First Affiliated Hospital of Harbin Medical University. The patients/participants provided their written informed consent to participate in this study. Written informed consent was obtained from the individual(s) for the publication of any potentially identifiable images or data included in this article.

## Author Contributions

QW, CL, HS, and PW contributed to study conception and design. QW, CL, SX, CW, ZJ, JQ, YL, and BS contributed to data acquisition and data interpretation. QW and CL contributed to the data analysis and drafted the manuscript. HS and PW contributed to the major revision of the manuscript. HS, PW, SX, CW, ZJ, and JQ contributed to the significant intellectual content. All authors have made a significant contribution to this study including, manuscript preparation, critically revised the article, and approved the final version of the manuscript.

## Funding

This study was supported by grants from the National Natural Science Foundation (81901190), the Natural Science Foundation of Heilongjiang Province of China (YQ2019H05), and Foundation of the First Affiliated Hospital of Harbin Medical University (2019L02).

## Conflict of Interest

The authors declare that the research was conducted in the absence of any commercial or financial relationships that could be construed as a potential conflict of interest.

## Publisher's Note

All claims expressed in this article are solely those of the authors and do not necessarily represent those of their affiliated organizations, or those of the publisher, the editors and the reviewers. Any product that may be evaluated in this article, or claim that may be made by its manufacturer, is not guaranteed or endorsed by the publisher.
